# Seasonal Investigation of *Anaplasma marginale* Infection in Pakistani Cattle Reveals Hematological and Biochemical Changes, Multiple Associated Risk Factors and *msp5* Gene Conservation

**DOI:** 10.3390/pathogens11111261

**Published:** 2022-10-29

**Authors:** Muhammad Asif, Mourad Ben Said, Rommel Lenin Vinueza, Renato Leon, Nadeem Ahmad, Asia Parveen, Adil Khan, Arusa Ejaz, Muhammad Ali, Asmat Ullah Khan, Muhammad Baber, Furhan Iqbal

**Affiliations:** 1 Institute of Molecular Biology and Biotechnology, Bahauddin Zakariya University Multan, Multan 60800, Pakistan; 2 Department of Basic Sciences, Higher Institute of Biotechnology of Sidi Thabet, University of Manouba, Manouba 2010, Tunisia; 3 Laboratory of Microbiology, National School of Veterinary Medicine of Sidi Thabet, University of Manouba, Manouba 2010, Tunisia; 4 Instituto de Medicina Social y Desafíos Globales, Colegio de Ciencias de la Salud, Universidad San Francisco de Quito USFQ, Quito 170901, Ecuador; 5 Laboratorio de Entomología Médica & Medicina Tropical LEMMT, Colegio de Ciencias Biológicas y Ambientales COCIBA, Universidad San Francisco de Quito USFQ, Quito 170901, Ecuador; 6 Center of Excellence in Molecular Biology, 87-West Canal Bank Road, Thokar Niaz Baig, Lahore 53700, Pakistan; 7 Institute of Zoology, Bahauddin Zakariya University Multan, Multan 60800, Pakistan; 8 Department of Botany and Zoology, Bacha Khan University, Charsadda 24420, Pakistan; 9 College of Veterinary Sciences, Bahauddin Zakariya University, Bahadur Sub Campus, Layyah 31200, Pakistan; 10 Shaheed Benazir Bhuto University Sheringal, District Dir (Upper), Khyber Pakhtunkhwa, Sheringal 18050, Pakistan

**Keywords:** *Anaplasma marginale*, seasonal survey, associated risk factors, hematological and biochemical changes, *msp5* gene conservation, Pakistani cattle

## Abstract

Bovine anaplasmosis is a tick-borne disease caused by an obligate intercellular Gram-negative bacterium named *Anaplasma* (*A.*) *marginale.* In this study, we report the seasonal prevalence, potentially associated risk factors and phylogeny of *A. marginale* in cattle of three different breeds from Multan District, Southern Punjab, Pakistan. A total of 1020 blood samples (crossbred, n = 340; Holstein Friesian, n = 340; and Sahiwal breed, n = 340) from apparently healthy cattle were collected on a seasonal basis from March 2020 to April 2021. Based on PCR amplification of the *msp5* partial sequence, overall, the *A. marginale* prevalence rate was estimated at 11.1% (113/1020) of the analyzed cattle samples. According to seasons, the highest prevalence rate was observed in autumn (16.5%), followed by winter (10.6%) and summer (9.8%), and the lowest was recorded in the spring (7.5%). The crossbred and Sahiwal cattle were the most susceptible to *A. marginale* infection, followed by Holstein Friesian cattle (7.9%). Analysis of epidemiological factors revealed that cattle reared on farms where dairy animals have tick loads, dogs coinhabit with cattle and dogs have tick loads have a higher risk of being infected with *A. marginale*. In addition, it was observed that white blood cell, lymphocyte (%), monocyte (%), hematocrit, mean corpuscular hemoglobin and mean corpuscular hemoglobin concentrations were significantly disturbed in *A. marginale*-positive cattle compared with non-infested cattle. Genetic analysis of nucleotide sequences and a phylogenetic study based on *msp5* partial sequencing demonstrated that this gene appears to be highly conserved among our isolates and those infecting apparently healthy cattle from geographically diverse worldwide regions. The presented data are crucial for estimating the risk of bovine anaplasmosis in order to develop integrated control policies against bovine anaplasmosis and other tick-borne diseases infecting cattle in the country.

## 1. Introduction

Pakistan is a country where agricultural production is highly dependent on rainfall and livestock provide financial prosperity in the event of poor harvests [1]. The majority of Pakistan’s population lives in rural areas, where livestock contribute significantly to the economy by providing valuable animal proteins in the form of milk and meat as well as providing skins and textile products [2]. As a result, this sector contributes significantly to Pakistan’s GDP. During the financial year 2020/2021, this industry contributed 11.5% of GDP [3]. In Pakistan, the cumulative dairy cattle population is 51.5 million head, providing an estimated 21,691 to 23,357 million tons of milk, placing Pakistan fourth in the world after China, India and the United States with respect to milk production [1].

Cattle diseases are a major constraint, as they pose financial risks for dairy farmers. Ectoparasites cause the majority of infections in cattle, and ticks are the most prevalent carriers of pathogens [4]. Ticks are well-known to have a detrimental influence on both wildlife and humans by infesting them and transmitting a variety of pathogens [5].

Tick-borne diseases remain an economic burden on the livestock industry in tropical and subtropical regions of the world [6]. In recent years, changes in seasonal activity caused by global warming and deforestation and the introduction of new animal species in various countries have favored increases in tick populations and in the pathogenicity of their transmitted bacteria, leading to increased economic losses [7].

Bovine anaplasmosis is an endemic tick-borne bacterial disease in tropical and subtropical areas caused mainly by *A. marginale*, an obligate intra-erythrocyte Gram-negative bacterium belonging to the order Rickettsiales [8,9]. This rickettsial agent is transmitted to animal hosts essentially by ticks, and 20 tick species have been experimentally confirmed as efficient vectors of *A. marginale* [10]. Indeed, ticks of *Ixodes* spp., *Dermacentor* spp., *Rhipicephalus* spp. and *Amblyomma* spp. are reported to be involved in the transmission of *A. marginale* [11]. Cattle from Pakistan have been investigated for the presence of *Anaplasma marginale* as well as for tick infestations. In a recent study, Ashraf et al. [1] reported a small number of ticks present on cattle, including *Hyalomma anatolicum*, *Hyalomma excavatum*, *Rhipicephalus microplus* and *Haemaphysalis punctata*, but none of these ticks was found to be PCR-positive for the presence of *A. marginale*. Major clinical signs include pyrexia, progressive anemia, jaundice, anorexia, depression, reduced milk production, abortion in pregnant animals and death, particularly in exotic breeds [12]. Anaplasmosis is considered a zoonotic problem. This means that it has the potential to infect humans. However, A. marginale has not been detected in humans, and direct transmission of *Anaplasma* spp. from animals to people or animal to animal is highly unlikely and has not been documented [13]. The control of bovine anaplasmosis is mainly carried out using antibiotics and the elimination of arthropod vectors. Animals infected with *Anaplasma* and treated with Oxytetracycline normally resolve infection [14].

Serological and molecular diagnoses are the main methods allowing the detection of *A. marginale*, as their sensitivity and specificity are high [15]. In particular, Polymerase Chain Reaction (PCR), based on the amplification of DNA fragments, has been recommended to detect infection in animals intended for international trade and/or movement [16,17]. PCR has been shown to be effective even for testing complex blood samples with hemolyzed and coagulated bovine blood. A positive result for an Enzyme-Linked Immunosorbent Assay (ELISA) confirms the presence of anti-*A. marginale*; however, it does not necessarily mean that the pathogen was present at the time the test was performed [15]. The unique gold standard remains xenodiagnosis, which is not very practical [18]. For this reason, PCR is recommended as a confirmatory test for the diagnosis of bovine anaplasmosis [19]. In addition to PCR, the sequencing of 16SrRNA, *msp4*, *msp5* and/or *groESL* partial sequences has been used in most cases in order to identify and confirm the genus and species of bacterial isolates [20,21].

Although Multan District is known for its large cattle farms and dairy products, this study is the first ever from this region to investigate *A. marginale* infection in cattle according to seasons and to report the association of this infection with epidemiological risk factors and hematological and biochemical changes.

## 2. Materials and Methods

### 2.1. Study Area, Sampling and Data Collection

**Figure 1:**
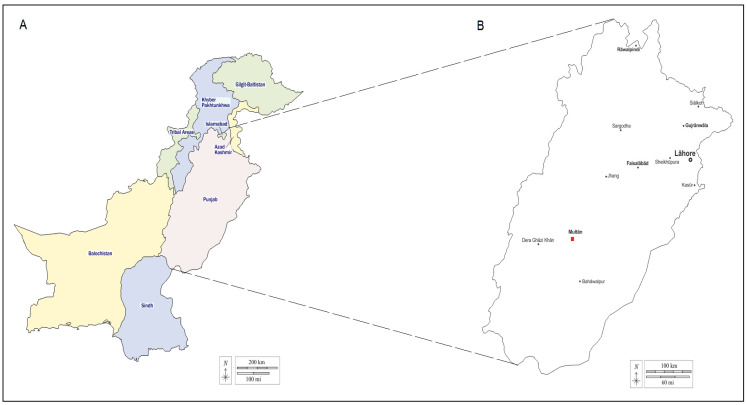
Map of the studied region in Pakistan. Legend: (**A**) Map of Pakistan showing Punjab Province, (**B**) Map of Punjab showing the location of Multan District, indicated by a red dot.

Multan is an ancient city in the Asian subcontinent located at latitude 30.2 N, 71.45 E and an altitude of 710 feet (Figure 1). It has an arid atmosphere, with a typical annual precipitation of 127 mm [22]. Summer is scorching and evident, and winter is short, cool and mostly clear. The temperature fluctuates from 43°F to 107°F throughout the year and is rarely below 38°F or above 113°F [23]. Blood samples from 1020 apparently healthy cattle (Holstein Friesian, Sahiwal and crossbred cattle) were collected from livestock farms (n = 48) in various areas of Multan District, Southern Punjab, Pakistan, on a seasonal basis from March 2020 to April 2021. Solvin’s formula was used to estimate the sample sizes that were randomly collected during the present study. Solvin’s formula was calculated as follows: n = N/(1 + N × e^2^), where n = number of samples, N = total population and e = margin of error. All the enrolled animals had no previous history of disease, and they were not suffering from any reproductive disorder at the time of sample collection. The seasons in which animal samples were collected were summer (May to July), autumn (August to October), winter (November to January) and spring (February to April). The dairy farms were diverse as far as the numbers and types of animals kept were concerned. Additionally, some farms used traditional methods of animal farming, while others were more organized, using modern tools to maintain hygiene and vaccinate cattle against tick-borne diseases. After the informed consent of the owners was obtained, the animals were examined by the veterinarian at the sampling sites and a blood sample (roughly 10 mL) was collected from the jugular vein of each animal and immediately sealed in a sterile tube containing 0.5 M EDTA as an anticoagulant, to be used for DNA extraction and for complete blood count analysis. A questionnaire was completed at the sampling site in order to collect data on epidemiological risk factors, such as gender, water source, tick load, other animals associated with the herd, dogs present in the herd and tick loads on dogs. All animal-handling procedures and laboratory protocols were approved by the ethical committee of the Institute of Molecular Biology and Biotechnology, Bahauddin Zakariya University Multan, Pakistan, under the application number IMBB/Ethics/2020-55.

### 2.2. DNA Extraction and PCR Amplification

Genomic DNA extraction was performed on the blood samples using an inorganic method according to Saeed et al. [24], and the DNA was stored at −20°C until use. A set of oligonucleotide primers, Fwd 5′ACAGGCGAAGAAGCAGACAT3′ and Rev 5′ATAAATGGGAACACGGTGGA 3′, was used to specifically amplify 382 base pairs of the *msp5* gene of *A. marginale* in positive blood samples [25]. The reaction mixture was prepared in a final volume of 25 µL, containing 13 mM Tris–HCl (pH 8.3), 65 mM KCl, 2 mM MgCl2, 0.0013% gelatin, 300 µM of each dNTP, 1 U of AmpliTaq DNA polymerase, 0.5 µM of each primer and 5 µL (50 to 150 ng) of template DNA. Reaction conditions included an initial denaturation step of 94 °C for 5 min, followed by 30 cycles of denaturation at 94 °C for 45 s, primer annealing at 53 °C for 50 s and extension at 72 °C for 50 s. A final extension at 72 °C for 7 min was performed [25]. *Anaplasma marginale*-positive and -negative samples were also added during each PCR reaction as positive and negative controls, respectively.

### 2.3. DNA Sequencing and Phylogenetic Analysis

Four representative PCR products were randomly selected, purified from agarose gel slices with NZYGelpure^®^ (Nzytech, Portugal) and subsequently sequenced (First Base Sequencing Service, Malaysia), with the same primers used for DNA amplification. The obtained DNA sequences from *A. marginale* isolates 101, 3, 20 and 67 were deposited with GenBank under the accession numbers MW759701-MW759704, respectively. DNAMAN software was also used to assess the variability of the nucleotide positions in the analyzed sequence alignments. BLAST analysis of GenBank was used to assess the level of similarity to previously reported sequences (http://blast.ncbi.nlm.nih.gov/, accessed on 20 September 2022) [26]. Using the same software, genetic distances among the operational taxonomic units were calculated by the maximum composite likelihood method [27] and were used to construct a neighbor-joining tree after the alignment of *A. marginale msp5* partial sequences [28]. The statistical support for internal branches of the phylogenetic tree was evaluated by bootstrapping with 1000 iterations [29].

### 2.4. Hematological Analysis

Complete blood count parameters in all cattle blood samples were analyzed using an automated complete blood count analyzer, as described by Ashraf et al. [1].

### 2.5. Statistical Analysis

All data with quantitative values are expressed as the means ± standard errors of the means. The statistical package Minitab (version 17, USA) was used for data statistical analysis. Associations between *A. marginale* occurrence and various risk factors, i.e., tick infestation, presence of dogs and other animals in the herd, and presence of dogs infested with ticks, were assessed by contingency table analysis using Fisher’s exact test (for 2 × 2 tables). Two sample *t*-tests were performed to compare the different studied hematological parameters between *A. marginale*-positive and -negative blood samples. Comparisons of *A. marginale* prevalence rates between sampling seasons and different cattle breeds were made using the chi-square (χ2) test. Significance levels were set at *p* = 0.05.

## 3. Results

### 3.1. Molecular Investigation and Risk Factor Analysis

Overall, the *A. marginale* prevalence rate was 11.1% (113/1020) in the tested cattle. Seasonal analysis indicated that the highest rate of *A. marginale* infection was observed in autumn (16.5, 42/255), followed by winter (10.6%, 27/255), summer (9.8%, 25/255) and spring (7.5%, 19/255) (Table 1, *p* = 0.009). Crossbred and Sahiwal cattle were found to be the most highly *A. marginale*-infected breeds (12.6%, 43/340), followed by Holstein Frisian cattle (12.6%, 27/340) (Table 1).

According to the breeds of cattle collected in each season, risk factor analysis showed that none of the studied epidemiological factors was found to be significantly associated with *A. marginale* infection during the four seasons for crossbred cattle (Table 2), while, in spring, Holstein Frisian cattle on farms where dogs were present (*p* = 0.013), especially those where the dogs were infested with ticks (*p* < 0.001), were statistically more infected with *A. marginale* than those raised with uninfested dogs (Table 3). In addition, in autumn, Holstein Frisian cattle infested with ticks (*p* = 0.001) and raised with other dairy animals (*p* = 0.023) were statistically more infected with *A. marginale* (Table 3). In autumn, cattle of the Sahiwal breed raised with dogs with tick loads had higher *A. marginale* infection rates (Table 4).

### 3.2. Complete Blood Count Analysis

For crossbred cattle, significant decreases in mean corpuscular hemoglobin concentration (*p* = 0.009) and platelet count (*p* = 0.006) during summer and in white blood cell count (*p* = 0.043), mean cell volume (*p* = 0.024) and mean cell hemoglobin (*p* = 0.036) during autumn were observed in *A. marginale*-positive blood samples compared with negative samples (Table 5). For Holstein Frisian cattle, significant decreases in monocyte percentages (*p* = 0.008) during spring and summer and in white blood cell (*p* = 0.007) and lymphocyte percentages (*p* = 0.023) during winter were observed, while increases in monocyte percentages (*p* = 0.0001) during summer and in platelet counts during spring (*p* = 0.017) were recorded in *A. marginale*-positive samples (Table 6). For cattle of the Sahiwal breed, the collected blood samples showed significant decreases in monocyte percentages (*p* = 0.02) during summer (Table 7).

### 3.3. Genotyping, Genetic Diversity Analysis and Phylogenetic Study

**Figure 2:**
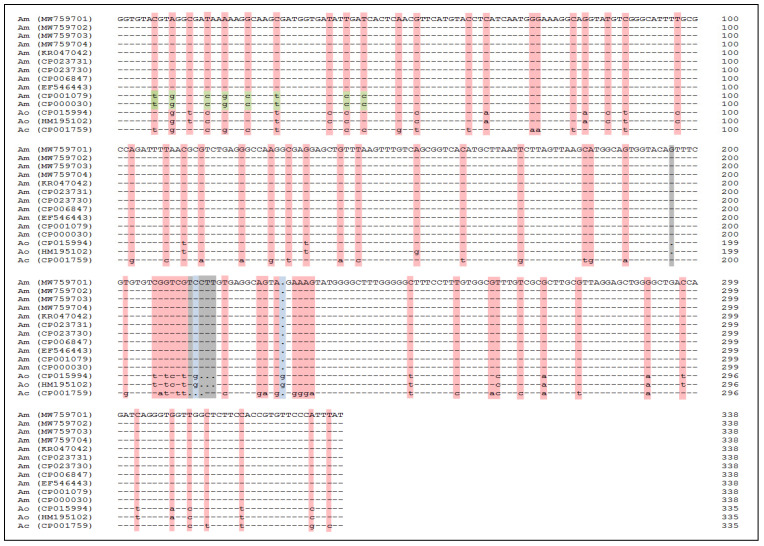
DNA sequence alignment based on partial *msp5* sequences from *Anaplasma marginale* isolates from cattle in Pakistan and all sequences representing worldwide *Anaplasma* spp. isolates available in GenBank covering more than 99% of the analyzed sequence.

Legend: Conserved nucleotide positions are indicated with dashes. The positions with deletions between *Anaplasma* spp. are represented by points and colored in gray, and the positions with substitutions between *Anaplasma* spp. are represented by different nucleotides and colored in pink. The positions with deletions and substitutions between *Anaplasma* spp. are colored in blue. The substituted nucleotides present in Florida and St. Maries strains (CP001079 and CP000030, respectively) compared with our sequences for *A. marginale* (MW759701-MW759704) are colored in green. Nucleotides: T, Thymine; C, Cytosine; G, Guanine; A, Adenine. Note: Information on the host, the strain or isolate name, and the country of origin of each sequence is presented in Figure 3.

The alignment of *msp5* partial sequences (341 bp) of four Pakistani isolates allowed the identification of a single genotype. The analysis of all partial *A. marginale* sequences available in GenBank covering the entire analyzed sequence revealed two genetic variants with eight nucleotide positions affected by substitutions (Figure 2). Indeed, we precisely selected these two genetic variants by the differentiation of one from the other according to the presence of at least one mutation in the nucleotide sequence which could be a substitution, a deletion or an addition (Figure 2). When we compared our four *msp5* partial sequences with other *A. marginale* sequences available in GenBank, the identity between sequences was found to range from 97.6 to 100%. By comparing our *msp5 A. marginale* partial sequences with sequences from *A. ovis* and *A. centrale*, which are genetically the closest species to *A. marginale*, the identity rates were estimated at 91.0 and 83.9%, respectively, noting the presence of nucleotide substitutions, deletions and additions (Figure 2).

**Figure 3:**
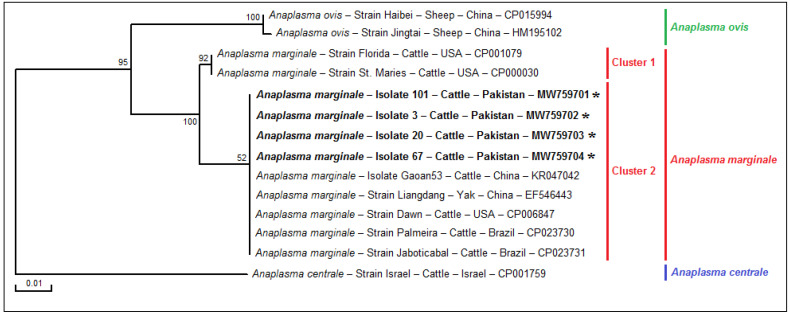
Phylogenetic tree based on *msp5* partial sequences (341 bp) from *Anaplasma marginale* isolates from cattle in Pakistan and those from all available worldwide isolates published in GenBank covering more than 99% of the analyzed sequence.

Legend: Bootstrap values (1000 replicates) are indicated in each node (only percentages greater than 50% are shown). The four *A. marginale* isolates obtained in the present study are represented in bold and indicated with asterisks. The hosts, the strain or isolate names, the countries of origin and the GenBank accession numbers are represented. Two *A. ovis* isolates (GenBank accession numbers CP015994 and HM195102) were added to the tree. One *A. centrale* isolate (GenBank accession number CP001759) was added as an out-group.

Phylogenetic analysis of the partial *msp5* (341 bp) gene was performed with sequences from this study and all available sequences from GenBank covering at least 99% of the analyzed sequence (Figure 3). According to the high rate of robustness nodes (100%), all *A. marginale* sequences clustered in two different clusters, as shown in Figure 3. The first cluster includes our four isolates clustered with those found in apparently healthy cattle and yak from the USA, Brazil and China. The second was formed according to partial sequences representing pathogenic strains infecting diseased cattle from the USA (Figure 3).

## 4. Discussion

Bovine anaplasmosis poses a serious challenge to profitable livestock production in tropical and subtropical countries [30]. It is caused mainly by *A. marginale* and represents an economic problem for cattle breeders due to the losses it generates, such as mortalities, reduced production, quarantine measures, treatments and control of vectors [9].

The present study was conducted to provide accurate information on the prevalence, potentially associated risk factors and genetic characteristics of *A. marginale* in cattle, which is useful for the design of cost-effective control measures. Recorded *A. marginale* prevalence was estimated at 11% in cattle located in Multan District, Pakistan (Table 1). In this country, there have been few other studies on the prevalence of *A. marginale* in cattle. Indeed, Zafar et al. [31] reported a prevalence of *Anaplasma marginale* estimated at 9% and 11% in cattle, respectively, from Lodhran and Dera Ghazi Khan Districts. In addition, Ashraf et al. [1] reported a rate of 8.6% in cattle blood samples collected from Layyah District in Punjab, Pakistan. Farooqi et al. [32] reported a higher prevalence rate estimated at 18.3% in bovine samples investigated in three distinct zones of KPK Province. In another study from KPK, Turi et al. [33] recorded a prevalence rate of 41.6% in cattle from Peshawar and Lakki Marwat Districts. Hussain et al. [2] reported that 6.1% of the apparently healthy animals of the Cholistan cattle breed analyzed in Bahawalpur District in Punjab were found to be infected with *A. marginale*. Prevalences of *A. marginale* infection in cattle have also been reported in several countries across the world, including Tunisia (25.4%), Turkey (29.1%), Brazil (27%) and the Galapagos Islands in Ecuador (100%) by Belkahia et al. [34], Zhou et al. [35], Vieira et al. [36] and Gioia [37], respectively. The variation in the prevalence of *A. marginale* in cattle from various countries, as has been made evident in this discussion, is due to the nature and magnitude of tick-control programs running in these countries and also due to the climatic and geographical differences that make these countries more or less suitable for ticks [34].

In this study, the highest prevalence of *A. marginale* in cattle was observed in the autumn season (Table 1). The higher prevalence of *A. marginale* in autumn months, when the tick vectors are not very common, suggests that mechanical transmission may play a role in the transmission of *A. marginale*. Our results are in agreement with those of Ashraf et al. [1], who also reported a highest prevalence of infection during the autumn season, probably due to the abundance of tick vectors showing high activity during the summer months. Nevertheless, our findings are contradictory to those of Roy et al. [38], who reported a highest prevalence in summer. This lets us suggest that the discrepancies in *A. marginale* prevalence rates between these studies are essentially due to the differences in geographical locations, abundances of tick vectors and breeding microenvironments [39].

In the present study, we have reported highest infection prevalence in Sahiwal breed cattle (Table 1). Our results contradict those of Ashraf et al. [1], which showed a highest *A. marginale* infection rate in Holstein Friesian cattle compared to crossbred and Sahiwal cattle. Similarly, Khan et al. [40] and Tay et al. [41] also reported higher levels of *Anaplasma* spp. prevalence rates in Holstein cattle compared with local breeds and suggested that the longer and thicker coat of this breed makes it a preferable host for ticks, while Zafar et al. [31] reported that no specific cattle breed was susceptible to *A. marginale* infection. These variations in *A. marginale* prevalence between cattle breeds observed in different studies are probably due to the differences in the numbers of tested samples. In fact, such factors can clearly affect the breed-specific prevalence ratios for *A. marginale* and probably for other tick-borne bacteria [1].

In Pakistan, it is common to keep watchdogs on livestock farms, and often these dogs are tick-infested too. In this survey, the presence of dogs and dogs with tick loads are risk factors significantly associated with bovine anaplasmosis (Table 2, Table 3 and Table 4). Dogs from Pakistan have been reported as infested with *Rh. sanguineus* s. l. and *Hyalomma anatolicum anatolicum*, which also infect small and large ruminants [21]. It is possible that physical contact between dogs and animals could cause tick transmission from one host to another, leading to the spread of tick-borne illnesses between them. Our findings are in agreement with those of Ashraf et al. [1], which indicated that dogs were carriers of ticks bringing *Anaplasma* infection to cattle herds. Our results are contradictory to those of Ashraf et al. [8], which showed a higher prevalence of *A. marginale* in buffalo herds without dogs. On traditionally managed farms, animals without regular treatment against tick-borne diseases are more frequently infected with *A. marginale* [1]. Swai et al. [42] and Atif et al. [43] also reported significantly higher prevalence of *A. marginale* on traditionally managed farms than on modern ones. In Pakistan, mismanaged and ancient traditional techniques for animal husbandry and management are still in practice, which leads to poor hygiene and increases the risk of tick infestation and tick-borne bacterial infections [31].

In this study, significant decreases in white blood cells (WBCs), lymphocytes (%) monocytes (%), platelet counts and hematocrit, mean cell hemoglobin and mean corpuscular hemoglobin concentrations were observed in infected cattle (Table 5, Table 6 and Table 7). Our results are in agreement with those of Ashraf et al. [1], which indicated a decrease in white blood cells, lymphocytes (%), monocytes (%) and hematocrit, mean corpuscular hemoglobin and mean corpuscular hemoglobin concentrations in *A. marginale*-positive cattle. Riond et al. [44] also reported a decrease in mean corpuscular hemoglobin concentration and an increase in mean corpuscular volume in cattle infected with *A. marginale*. In effect, the decreases in hematocrit, mean cell hemoglobin and mean corpuscular hemoglobin concentrations are due to the fact that *A. marginale* invades red blood cells (RBCs) during infection [1]. Rapid RBC destruction by phagocytosis leads to their increased demand and bone marrow cells begin to release larger immature RBCs with higher corpuscular volumes than mature RBCs [25,44]. Our results coincide with those of Khan et al. [40], who recorded a significant decrease in WBCs in the pre-patent phase and a non-significant decrease in WBCs in early and late stages of the disease. Similarly, Zafar et al. [31] also showed a significant decrease in white blood cells (WBCs), platelet counts and lymphocytes (%) in *A. marginale*-positive cattle. Riond et al. [44] demonstrated that anemic cattle had thrombocytopenia (low blood platelet counts). This increase in platelet consumption may be attributed to disseminated intravascular coagulation [45].

In this study, genetic analysis of nucleotide sequences and a phylogenetic study based on *msp5* partial sequences demonstrated that this gene appears to be highly conserved among our isolates and those infecting apparently healthy cattle and yak from geographically diverse regions worldwide. Phylogenetic analysis revealed that our amplified DNA sequences form a stable monophyletic cluster (the second cluster), with 100% homology with *msp5* partial sequences from China, the USA and Brazil (Figure 3). This finding is in agreement with those of Zafar et al. [31], who reported that sequences of *A. marginale* were clustered with previously reported *A. marginale* sequences infecting apparently healthy cattle from Pakistan (MK032842-3), South Africa (KU647713 and -14 and KU647716) and Israel (AY841153). However, our isolates are relatively distant from the highly pathogenic Florida and St. Maries strains isolated from diseased cattle in the USA. The genetic variations in *msp5* sequences of *A. marginale* observed during this analysis are probably due to differences in geographic conditions which cause the appearance of different tick vectors with various *A. marginale* strains with different levels of pathogenicity [17,46].

## 5. Conclusions

We have reported here, for the first time in Pakistan, the seasonal dynamics of *A. marginale* infection in three cattle breeds from Multan District in Punjab. Crossbred and Sahiwal cattle were the most susceptible to *A. marginale*, and the highest bacterial infection rates were observed during the autumn season. Cattle raised on farms where other animals and dogs were kept with cattle were more susceptible to *A. marginale* infection. Further studies are needed to better characterize the different isolates of this species using more discriminative genes and to identify the main vectors implicated in the transmission of this *Anaplasma* species in Pakistan.

## Figures and Tables

**Table 1:** Overall and seasonal prevalences of *Anaplasma marginale* in blood samples of Sahiwal, Holstein Friesian and crossbred Pakistani cattle included in the present study.

Season	*A. marginale*-Positive Samples/Total (% ± C.I. ^1^)				
	Crossbred	Holstein Friesian	Sahiwal	Total	*p*-Value ^2^
Spring (n = 255)	5/85 (5.9 ± 0.05)	6/85 (7.1 ± 0.01)	8/85 (9.4 ± 0.02)	19/255 (7.5 ± 0.03)	0.671
Summer (n = 255)	8/85 (9.4 ± 0.02)	6/85 (7.1 ± 0.01)	11/85 (12.9 ± 0.07)	25/255 (9.8 ± 0.03)	0.419
Autumn (n = 255)	18/85 (21.2 ± 0.08)	9/85 (10.6 ± 0.06)	15/85 (17.6 ± 0.08)	42/255 (16.5 ± 0.04)	0.165
Winter (n = 255)	12/85 (14.1 ± 0.07)	6/85 (7.1 ± 0.01)	9/85 (10.6 ± 0.06)	27/255 (10.6 ± 0.03)	0.326
Total (n = 1020)	43/340 (12.6 ± 0.03)	27/340 (7.9 ± 0.02)	43/340 (12.6 ± 0.03)	113/1020 (11.1 ± 0.01)	0.078
*p*-value ^3^	0.017 *	0.780	0.382	0.009 *	-

Abbreviations: ^1^: C.I.: 95% confidence interval; ^2^: *p*-values were calculated according to breeds for the same season. ^3^: *p*-values were calculated according to seasons for the same breed. *: Statistically significant test.

**Table 2:** Prevalence rates of *Anaplasma marginale* in crossbred cattle analyzed in four seasons according to different risk factors related to animals and herds.

Rick Factors	Classes	*A. marginale*-Positive Samples/Total (Infection Rate (%) ± C.I. ^1^)							
		Spring	*p*-Value	Summer	*p*-Value	Autumn	*p*-Value	Winter	*p*-Value
Tick loads on cattle	Present	2/45 (4.4 ± 0.06)	0.552	8/80 (10 ± 0.06)	0.460	14/69 (20.3 ± 0.09)	0.679	11/61 (18.0 ± 0.09)	0.100
	Absent	3/40 (7.5 ± 0.08)		0/5 (0)		4/16 (25 ± 0.21)		1/24 (4.2 ± 0.08)	
Other dairy animals at farm	Present	1/42 (2.4 ± 0.04)	0.177	6/43 (13.9 ± 0.10)	0.149	11/62 (17.7 ± 0.09)	0.205	5/36 (13.9 ± 0.11)	0.958
	Absent	4/43 (9.3 ± 0.08)		2/42 (4.8 ± 0.06)		7/23 (30.4 ± 0.18)		7/49 (14.3 ± 0.09)	
Dogs at farm	Present	4/54 (7.4 ± 0.07)	0.433	6/70 (8.5 ± 0.06)	0.568	14/53 (26.4 ± 0.11)	0.255	10/64 (15.6 ± 0.08)	0.488
	Absent	1/31 (3.2 ± 0.06)		2/15 (20.0 ± 0.20)		4/32 (12.5 ± 0.11)		2/21 (9.5 ± 0.12)	
Tick loads on dogs	Present	2/15 (13.3 ± 0.17)	0.179	6/60 (10 ± 0.07)	0.774	11/40 (27.5 ± 0.13)	0.181	11/66 (16.7 ± 0.09)	0.211
	Absent	3/70 (4.3 ± 0.04)		2/25 (8.0 ± 0.10)		7/45 (15.6 ± 0.10)		1/19 (5.3 ± 0.09)	
Total		5/85 (5.9 ± 0.05)		8/85 (9.4 ± 0.06)		18/85 (21.2 ± 0.08)		12/85 (14.1 ± 0.07)	

Abbreviations: ^1^: C.I.: 95% confidence interval.

**Table 3:** Prevalence rates of *Anaplasma marginale* in Holstein Friesian cattle analyzed in four seasons according to different risk factors related to animals and herds.

Rick Factors	Classes	*A. marginale*-Positive Samples/Total (Infection Rate (%) ± C.I. ^1^)							
		Spring	*p*-Value	Summer	*p*-Value	Autumn	*p*-Value	Winter	*p*-Value
Tick loads on cattle	Present	2/30 (6.7 ± 0.09)	0.917	2/34 (5.8 ± 0.07)	0.731	6/20 (30.0 ± 0.19)	0.001 *	5/57 (8.8 ± 0.07)	0.381
	Absent	4/55 (7.3 ± 0.06)		4/51 (7.8 ± 0.07)		3/65 (4.6 ± 0.05)		1/28 (3.6 ± 0.06)	
Other dairy animals at farm	Present	1/6 (16.7 ± 0.29)	0.343	4/68 (5.9 ± 0.05)	0.399	6/28 (21.4 ± 0.15)	0.023 *	5/62 (8.1 ± 0.06)	0.554
	Absent	5/79 (6.3 ± 0.05)		2/17 (11.8 ± 0.15)		3/57 (5.3 ± 0.05)		1/23 (4.3 ± 0.08)	
Dogs at farm	Present	5/31 (16.1 ± 0.12)	0.013 *	4/51 (7.8 ± 0.07)	0.731	8/51 (15.7 ± 0.09)	0.062	6/71 (8.4 ± 0.06)	0.262
	Absent	1/54 (1.8 ± 0.03)		2/34 (5.9 ± 0.07)		1/34 (2.9 ± 0.05)		0/14 (0)	
Tick loads on dogs	Present	4/11 (36.4 ± 0.04)	0.000 *	4/51 (7.8 ± 0.07)	0.731	6/32 (18.7 ± 0.13)	0.058	5/57 (8.8 ± 0.07)	0.381
	Absent	2/74 (2.7 ± 0.03)		2/34 (5.9 ± 0.07)		3/53 (5.7 ± 0.06)		1/28 (3.6 ± 0.06)	
Total		6/85 (7.1 ± 0.05)		6/85 (7.1 ± 0.05)		9/85 (10.6 ± 0.07)		6/85 (7.1 ± 0.05)	

Abbreviations: ^1^: C.I.: 95% confidence interval; *: Statistically significant test.

**Table 4:** Prevalence rates of *Anaplasma marginale* in Sahiwal cattle analyzed in four seasons according to different risk factors related to animals and herds.

Rick Factors	Classes	*A. marginale*-Positive Samples/Total (Infection Rate (%) ± C.I. ^1^)							
		Spring	*p*-Value	Summer	*p*-Value	Autumn	*p*-Value	Winter	*p*-Value
Tick loads on cattle	Present	7/63 (11.1 ± 0.07)	0.366	10/59 (16.9 ± 0.09)	0.099	14/80 (17.5 ± 0.08)	0.214	9/74 (12.2 ± 0.07)	0.223
	Absent	1/22 (4.5 ± 0.08)		1/26 (3.8 ± 0.07)		2/5 (40 ± 0.42)		0/11 (0)	
Other dairy animals at farm	Present	7/72 (9.7 ± 0.06)	0.818	11/69 (15.9 ± 0.08)	0.088	14/66 (21.2 ± 0.09)	0.296	5/48 (10.4 ± 0.08)	0.953
	Absent	1/13 (7.7 ± 0.14)		0/16 (0)		2/19 (10.5 ± 0.13)		4/37 (10.8 ± 0.09)	
Dogs at farm	Present	4/44 (9.1 ± 0.08)	0.916	10/72 (13.8 ± 0.08)	0.542	14/78 (17.9 ± 0.08)	0.493	6/50 (12.0 ± 0.09)	0.615
	Absent	4/41 (9.8.2 ± 0.09)		1/13 (7.7 ± 0.14)		2/7 (28.0 ± 0.33)		3/35 (8.6 ± 0.09)	
Tick loads on dogs	Present	3/34 (8.8 ± 0.09)	0.880	10/62 (16.1 ± 0.09)	0.152	11/78 (14.1 ± 0.07)	0.000 *	5/38 (13.2 ± 0.10)	0.491
	Absent	5/51 (9.8 ± 0.08)		1/23 (4.3 ± 0.08)		5/7 (71.4 ± 0.33)		4/47 (8.5 ± 0.08)	
Total		8/85 (9.4 ± 0.06)		11/85 (12.9 ± 0.07)		16/85 (18.8 ± 0.08)		9/85 (15.3 ± 0.06)	

Abbreviation: ^1^: C.I.: 95% confidence interval, *: Statistically significant test.

**Table 5:** Comparison of the analyzed complete blood count parameters between *Anaplasma marginale*-positive and -negative blood samples of crossbred cattle collected from Multan District over four seasons in 2020–2021.

Parameters	Spring		Summer		Autumn		Winter	
	*A. marginale*-Positive (n = 05)	*A. marginale*-Negative (n = 80)	*A. marginale*-Positive (n = 08)	*A. marginale*-Negative (n = 77)	*A. marginale*-Positive (n = 18)	*A. marginale*-Negative (n = 67)	*A. marginale*-Positive (n = 12)	*A. marginale*-Negative (n = 73)
White blood cells	11.04 ± 2.0	10.37 ± 0.50	7.70 ± 0.71	8.90 ± 0.82	8.39 ± 0.55	11.4 ± 1.4 *	8.5 ± 0.29	10.27 ± 0.79 *
Lymphocytes (%)	54.4 ± 9.2	56.1 ± 2.4	76.88 ± 1.9	75.55 ± 1.1	27.6 ± 3.5	23.4 ± 1.4	36 ± 3.8	38.8 ± 1.7
Monocytes (%)	4.7 ± 0.4	5.89 ± 0.5	4.37 ± 0.26	4.75 ± 0.48	3.06 ± 0.13	3.15 ± 0.05	4.7 ± 0.63	7.2 ± 0.79 **
Red blood cells	5.7 ± 0.4	6 ± 0.2	4.64 ± 0.36	5.19 ± 0.26	4.356 ± 0.20	4.396 ± 0.096	4.9 ± 0.17	5 ± 0.13
Hemoglobin	8.9 ± 1.0	9.17 ± 0.19	8.86 ± 0.47	9.35 ± 0.16	11.04 ± 0.33	11.04 ± 0.22	9.1 ± 0.3	8.9 ± 0.2
Mean cell volume	43.42 ± 1.1	43.66 ± 0.55	75.9 ± 4.9	70.9 ± 2.4	78.28 ± 1.5	82.27 ± 0.79 **	74.75 ± 2.4	71.9 ± 1.7
Hematocit	24.74 ± 1.9	26.59 ± 0.55	36.1 ± 3.8	32.23 ± 0.77	34.33 ± 1.5	35.43 ± 0.83	35.1 ± 1.1	38.4 ± 1 *
Mean cell volume	15.24 ± 0.86	14.84 ± 0.17	20 ± 2.1	19.05 ± 0.69	27.61 ± 0.52	28.97 ± 0.33 *	29.6 ± 0.5	28.4 ± 0.7
MCHC	36.60 ± 2.3	34.13 ± 0.42	26.5 ± 1.1	30.24 ± 0.54 **	33.44 ± 0.44	33.40 ± 0.30	33.3 ± 1.1	33.7 ± 0.4
Platelets	291 ± 53	246 ± 16	146.6 ± 26	247 ± 14 **	243.6 ± 18	240.4 ± 12	268.4 ± 13	274.8 ± 10

Abbreviations: Data are expressed as means ± standards errors of the means. The *p*-values indicate the results of two sample *t*-tests calculated for each studied parameter. *p* > 0.05 = Non-significant; *p* < 0.05 = Least significant (*); *p* < 0.01 = Significant (**). MCHC: Mean corpuscular hemoglobin concentration.

**Table 6:** Comparison of the analyzed complete blood count parameters between *Anaplasma marginale*-positive and -negative blood samples of Holstein Friesian cattle collected from Multan District during four seasons in 2020–2021.

Parameters	Spring		Summer		Autumn		Winter	
	*A. marginale*-Positive (n = 06)	*A. marginale*-Negative (n = 79)	*A. marginale*-Positive (n = 06)	*A. marginale*-Negative (n = 79)	*A. marginale*-Positive (n = 09)	*A. marginale*-Negative (n = 76)	*A. marginale*-Positive (n = 06)	*A. marginale*-Negative (n = 79)
White blood cells	10.2 ± 0.5	10.1 ± 0.3	8.0 ± 0.9	8.7 ± 0.3	9.8 ± 0.7	10.8 ± 0.3	8.1 ± 0.3	11 ± 0.9 **
Lymphocytes (%)	44.2 ± 4.3	47.8 ± 1.6	45.7 ± 4.5	53.2 ± 1.3	39.6 ± 4.8	45.7 ± 2.2	23.2 ± 4.9	39.1 ± 1.8 *
Monocytes (%)	3.7 ± 0.5	5.7 ± 0.3 **	1.7 ± 0.1	0.87 ± 0.1 ***	2.4 ± 0.6	2.3 ± 0.2	8.2 ± 4.4	7.3 ± 0.7
Red blood cells	5.8 ± 0.3	5.9 ± 0.1	5.02 ± 0.3	5.3 ± 0.1	5 ± 0.4	5 ± 0.1	4.5 ± 0.4	5 ± 0.1
Hemoglobin	9.9 ± 0.7	9.5 ± 0.2	8.1 ± 0.4	8.2 ± 0.2	8.9 ± 0.3	8.6 ± 0.2	9 ± 0.2	8.9 ± 0.1
Mean cell volume	43.2 ± 1.8	44.6 ± 0.5	47 ± 2	46.4 ± 0.7	54.6 ± 6.1	55.1 ± 2.1	69.2 ± 5.4	72.6 ± 1.6
Hematocit	24.8 ± 1.2	26 ± 0.4	24.8 ± 1.7	24.3 ± 0.6	26.9 ± 1.3	25.6 ± 0.6	34.1 ± 4.3	38.2 ± 1
Mean cell hemoglobin	17.1 ± 0.7	16.2 ± 0.2	14.7 ± 0.3	14.7 ± 0.1	18.4 ± 2	18.6 ± 0.7	28.2 ± 3.1	28.5 ± 0.7
MCHC	39.9 ± 2.0	36.6 ± 0.4	33 ± 1.3	33 ± 0.4	34.1 ± 0.7	34.6 ± 0.5	31.4 ± 1.2	33.5 ± 0.3
Platelets	465 ± 36	291 ± 13 **	296 ± 33	290 ± 13	263 ± 29	268 ± 16	276 ± 10	265 ± 6.9

Abbreviations: Data are expressed as means ± standard errors of the means. The *p*-values indicate the results of two sample *t*-tests calculated for each studied parameter. *p* > 0.05 = Non-significant; *p* < 0.05 = Least significant (*); *p* < 0.01 = Significant (**); *p* < 0.001 = Highly significant (***). MCHC: Mean corpuscular hemoglobin concentration.

**Table 7:** Comparison of analyzed complete blood count parameters between *Anaplasma marginale*-positive and -negative blood samples of Sahiwal cattle collected from Multan District during four seasons in 2020–2021.

Parameters	Spring		Summer		Autumn		Winter	
	*A. marginale*-Positive (n = 08)	*A. marginale*-Negative (n = 77)	*A. marginale*-Positive (n = 11)	*A. marginale*-Negative (n = 74)	*A. marginale*-Positive (n = 16)	*A. marginale*-Negative (n = 69)	*A. marginale*-Positive (n = 09)	*A. marginale*-Negative (n = 76)
White blood cells	8.48 ± 0.88	9.21 ± 0.30	7.65 ± 0.45	7.91 ± 0.26	9.16 ± 0.87	10.58 ± 1.1	10.99 ± 2.8	9.57 ± 0.67
Lymphocytes (%)	40.3 ± 5.3	40.3 ± 1.9	75.09 ± 1.8	77.24 ± 0.96	31.5 ± 4.1	25.5 ± 1.4	43 ± 5.7	47.8 ± 1.7
Monocytes (%)	5.99 ± 0.81	5.63 ± 0.34	3.18 ± 0.12	3.98 ± 0.33 *	3.07 ± 0.07	3.1 ± 0.03	5.03 ± 1.2	4.69 ± 0.48
Red blood cells	5.84 ± 0.32	6.46 ± 0.42	4.31 ± 0.28	4.54 ± 0.15	4.04 ± 0.14	4.2 ± 0.08	4.94 ± 0.92	4.96 ± 0.17
Hemoglobin	9.13 ± 0.33	9.18 ± 0.12	9.13 ± 0.33	9.32 ± 0.14	9.59 ± 0.48	10.48 ± 0.26	9.74 ± 0.89	9.29 ± 0.13
Mean cell volume	42.1 ± 1.2	43.34 ± 0.82	79.7 ± 3.8	77.1 ± 2.0	76.87 ± 1.6	80.41 ± 0.85	72.5 ± 4.3	74.6 ± 1.6
Hematocit	24.57 ± 1.5	26.33 ± 0.47	31.6 ± 1.5	31.1 ± 0.77	29.93 ± 1.4	32.76 ± 0.85	37.89 ± 2.7	40.77 ± 0.76
Mean cell hemoglobin	25.4 ± 10	16.08 ± 0.94	24.1 ± 0.17	17.7 ± 0.15	26.93 ± 1.1	27.34 ± 0.40	28.60 ± 1.9	29.63 ± 0.91
MCHC	37.75 ± 2.7	34.46 ± 0.63	29.8 ± 1.1	30.65 ± 0.46	33.27 ± 0.42	33.41 ± 0.22	33.58 ± 1.3	34.33 ± 0.31
Platelets	287 ± 23	250 ± 13	216 ± 26	258 ± 32	233 ± 19	242.2 ± 8.2	245 ± 26	279 ± 8.4

Abbreviations: Data are expressed as means ± standard errors of the means. The *p*-values indicate the results of two sample *t*-tests calculated for each studied parameter. *p* > 0.05 = Non-significant; *p* < 0.05 = Least significant (*). MCHC: Mean corpuscular hemoglobin concentration.

## Data Availability

All data that were generated or analyzed during this study are included in the article.
